# Ascorbic acid improves pluripotency of human parthenogenetic embryonic stem cells through modifying imprinted gene expression in the Dlk1-Dio3 region

**DOI:** 10.1186/s13287-015-0054-9

**Published:** 2015-04-14

**Authors:** Yang Yu, Qian Gao, Hong-cui Zhao, Rong Li, Jiang-man Gao, Ting Ding, Si-yu Bao, Yue Zhao, Xiao-fang Sun, Yong Fan, Jie Qiao

**Affiliations:** Department of Obstetrics and Gynecology, Center of Reproductive Medicine, Peking University Third Hospital, No. 49 HuaYuan North Road, HaiDian District, Beijing, 100191 People’s Republic of China; Key Laboratory of Assisted Reproduction, Ministry of Education, Beijing, 100191 China; Beijing Key Laboratory of Reproductive Endocrinology and Assisted Reproductive Technology, Beijing, 100191 China; Key Laboratory for Major Obstetric Diseases of Guangdong Province, the Third Affiliated Hospital of Guangzhou Medical University, No. 63, Liwan District, Guangzhou City, 510150 Guangdong Province People’s Republic of China

## Abstract

**Introduction:**

Human parthenogenetic embryonic stem cells (hpESCs) are generated from artificially activated oocytes, however, the issue of whether hpESCs have equivalent differentiation ability to human fertilized embryonic stem cells remains controversial.

**Methods:**

hpESCs were injected into male severe combined immunodeficiency (SCID) mice and the efficiency of teratoma formation was calculated. Then the gene expression and methylation modification were detected by real time-PCR and bisulfate methods.

**Results:**

Comparison of five hpESCs with different differentiation abilities revealed that levels of paternal genes in the Dlk1-Dio3 region on chromosome 14 in the hpESCs with high differentiation potential are enhanced, but strictly methylated and silenced in the hpESCs with lower differentiation potential. Treatment with ascorbic acid, rescued their ability to support teratoma formation and altered the expression profiles of paternally expressed genes in hpESCs that could not form teratoma easily. No differences in the expression of other imprinting genes were evident between hpESCs with higher and lower differentiation potential, except for those in the Dlk1-Dio3 region.

**Conclusions:**

The Dlk1-Dio3 imprinting gene cluster distinguishes the differentiation ability of hpESCs. Moreover, modification by ascorbic acid may facilitate application of hpESCs to clinical settings in the future by enhancing their pluripotency.

**Electronic supplementary material:**

The online version of this article (doi:10.1186/s13287-015-0054-9) contains supplementary material, which is available to authorized users.

## Introduction

Human embryonic stem cells (hESCs) have tremendous potential in regenerative medicine and cell therapy, as they can differentiate into cell derivatives of all three primary germ layers, endoderm, mesoderm and ectoderm. To obtain clinically applicable hESCs, parthenogenetic activation methods are preferred to the conventional method requiring oocyte fertilization. However, application of established cloned hESCs to the clinic is difficult at present because the hemagglutinating virus of Japan is used in all successful cases [1,2], whereas the hemagglutinating virus of Japan is not allowed to be used in the clinic at present. Therefore, parthenogenetic ES cells (pESCs) that were from parthenogenetic activated oocytes using an artificial method permitted in clinic settings [3], may be more feasible for clinical application.

pECSs have been derived from mouse [4], rabbit [5], monkey [6] and human sources [7-10], and their ability to differentiate into neurons and endoderm cells has been widely confirmed. Imbalance of imprinting gene expression and unique epigenetic modifications in the whole genome is reported to impair the pluripotency of pESCs. Allen and co-workers reported varying differentiation abilities of mouse pESCs, with some contributing to generation of mice after injection into tetraploid blastocysts and others failing to form specific cell tissues, particularly for skeletal muscle [11]. Vassena *et al.* suggested that loss of differentiation ability is easier during serial differentiation and reprogramming processes for human pESCs (hpESCs) [12]. Furthermore, in a study by Mai and co-workers, one of the hpESC lines failed to form derivatives from three germ layers, consistent with the suggestions of Lu *et al.* [13] and Liu *et al.* [14]. Clearly, defects in differentiation ability of hpESCs limit their clinical application.

The Dlk1-Dio3 genomic regions were evolutionarily conserved in mammals. This region includes the paternally expressed genes Dlk1, Rtl1, and Dio3, and maternally expressed genes Meg3, Meg8, and antisense Rtl1. In mouse, aberrant silencing of the imprinted Dlk1-Dio3 gene cluster was observed in most induced pluripotent stem cell (iPSC) clones that contributed poorly to chimeras and failed to support the development of iPSC-derived animals [15,16]. Subsequently, Stadtfeld *et al*. demonstrated that ascorbic acid attenuates hypermethylation of the Dlk1-Dio3 cluster, consequently enhancing the pluripotency of iPSCs reprogrammed from differentiated B cells [17]. Further studies by Bryce *et al*. suggested that aberrant DNA methylation within the Dlk1-Dio3 locus decreases the efficiency of generating mice via tetraploid complementation, supporting the contribution of this locus to the formation of low-quality iPSCs [18]. In addition to iPSCs, activation of paternally expressed imprinted genes in mouse pESCs enhances their pluripotency, even resulting in the production of germline-competent mice [19]. Additionally, expression profiling of the Dlk1-Dio3 cluster is correlated with pluripotency of iPSCs in pigs [20].

While studies on the Dlk1-Dio3 imprinting domain in hESCs have mainly focused on the field of tumor promotion, limited reports to date have validated the significance of this domain gene locus. Xie *et al*. showed that silencing of the Dlk1-Dio3 cluster of hESCs impairs generation of their differentiated hepatocyte-like cell derivatives but does not compromise multilineage differentiation ability [21]. Furthermore, the group of Sun revealed that the conserved miRNA cluster within the imprinted Dlk1-Dio3 region is highly expressed in mouse and rhesus macaque ESCs but rarely in hESCs [22]. Therefore, determination of expression patterns of the Dlk1-Dio3 cluster in hpESCs and analysis of its role in pluripotency regulation of hpESCs is necessary.

In the present study, we found that the Dlk1-Dio3 gene locus in hpESCs is aberrantly expressed and methylated, which correlated with teratoma formation. Furthermore, we examined the effects of ascorbic acid on methylation and gene expression patterns in the Dlk1-Dio3 cluster, with a view to ascertaining whether this compound contributes to improvement of the differentiation ability of hpESCs.

## Methods

### Ethical approval

The current study was approved by the Institutional Review Board of Peking University Third Hospital and Guangzhou Medical University Affiliated Third Hospital. The volunteers involved in the present study were informed of all details of the procedure, including sample utility and research destination, and voluntarily signed an informed consent document. All necessary consents from any participatant involved in the study, including consent to participate in the study, were obtained.

### Construction and culture of human fertilized and parthenogenetic embryos

Immature oocytes were collected from patients in the ICSI cycle because of male factor infertility in the reproductive medical centers at both Peking University Third Hospital and Guangzhou Medical University Affiliated Third Hospital. All immature oocytes were cultured in commercial *in vitro* maturation medium (SAGE In Vitro Fertilization, Inc., San Clemente, California, U.S.), and those that expelled the first polar body were regarded as mature oocytes at the metaphase II stage. One portion of MII oocytes was subjected to intra-cytoplasmic sperm injection (ICSI) micromanipulation to construct fertilized embryos and the other portion artificially activated by treatment for five minutes with 5 μM ionomycin and for two hours with 2 mM 6- dimethylaminopyridine (6-DMAP) to form parthenogenetic embryos. All embryos were cultured in G-1 medium, followed by G-2 medium from the eight-cell to the blastocyst stages (G1 and G2 medium were all purchased from Vitrolife Inc., Goteborg, Sweden).

### Derivation of hESCs from fertilized and parthenogenetic activated embryos

Expanded blastocysts were removed of zona pellucida using Tyrode’s solution, and the inner cell mass isolated via immunosurgery and placed on mitomycin C-treated murine embryonic fibroblast (MEF) feeder layers. After 7 to 10 days, the primary colony was transferred to a new feeder layer and culture continued for 10 more days. The ESC colony was dissected into four to eight pieces upon passage and implanted into new feeder layers. Two hpESCs and three human fertilized embryonic stem cell (hfESCs) lines were derived sucessfully. The former was designated as BGHPES-1 and BGHPES-2, and the latter was designated as BGHFESC-1, BGHFES-2 and BGHFES-3, which were also named FHES-CONT.

### General characterization of human embryonic stem cells

Characteristics of hESCs were determined using established protocols [23], as follows:

#### Alkaline phosphatase (AP) activity

AP activity was assessed in both hfESCs and hpESCs. Cell colonies were stained with BCIP/NBT for 8 to 12 minutes and examined under an inverted microscope.

#### Immunofluorescence staining

hESC colonies were removed to a glass sheet and fixed using 4% paraformaldehyde for 30 minutes. Fixed ESC colonies were permeabilized with 0.2% Triton X-100 for 30 minutes, followed by blocking in 3% BSA in PBS for 2 hours. Subsequently, colonies were incubated with 1:100 diluted primary antibodies, those specific for stem cell pluripotent markers (OCT4 and NANOG) (OCT4: 1:200, ab27985, Abcam, Cambridge, UK; NANOG: ab80892, Abcam) and stem cell surface markers (TRA-1-60) (1:200, ab16288, Abcam), and stained with the corresponding secondary antibodies, followed by treatment with 10 μg/mL propidium iodide for 30 minutes. Fluorescence was assessed under a confocal microscope (A1R, Nikon Corp., Tokyo, Japan).

#### Karyotyping

hESC colonies were implanted onto matrigel without a feeder layer, and cells collected after three-days culture. Next, colonies were rinsed in PBS solution and incubated in 0.075 mol/L potassium chloride for 10 minutes at 37°C. Finally, cells were fixed with methanol/glacial acetic acid and dropped onto glass slides. Chromosome spreads were Giemsa-banded and photographed. The karyotypes of hESCs were determined every 10 passages.

#### Differentiation in vitro and in vivo

hESCs were differentiated into embryoid bodies (EBs) *in vitro* by suspension culture. After 10 to 14 days, EBs were collected, and gene expression analyzed using RT-PCR methods. Specific genes in all three embryonic germ layers, including AFP (endoderm) (1:100, Human Germ Layer Marker Kit, Chemicon, Merck Millipore Corporation. The United States, Massachusetts city), SMA(mesoderm) (1:200, Human Germ Layer Marker Kit, Chemicon) and TUBULIN (ectoderm) (1:200, Human Germ Layer Marker Kit, Chemicon), were identified using immunofluorescence, and Nf68kd (endoderm), Hbz (mesoderm) and Albumin (ectoderm) with RT-PCR. Gene primers are listed in Additional file 1.

For *in vivo* differentiation, hESCs were injected into the inguinal grooves of six-week-old male severe combined immunodeficiency (SCID) mice after division into 300 to 400 small colonies. After two months, tumors were examined. All tumors were fixed for four to eight hours in 4% paraformaldehyde and embedded in paraffin. Sections were subsequently stained with hematoxylin and eosin, and examined under a light microscope for the presence of tissue derived from the three germ layers. In cases where tumors did not form within the two-month period, an equivalent volume of hESC colonies was injected into the same location. After three repeats of this step, hESCs were considered non-pluripotent.

### Gene expression profile analysis

Total RNA was extracted and purified using TRIzol with an established kit, and cDNA was generated from extracted RNA using reverse transcription. PCR was carried out using primers for the paternally expressed genes, Dlk1, Rtl1, and Dio3, and the maternally expressed genes, Meg3, Meg8, and antisense Rtl1 encoding miR433 and miR127. Amplified products were analyzed on a 1.5% agarose gel, visualized via ethidium bromide (Invitrogen, Life Technologies Corporation., Carlsbad, Calfornia, US) staining, and imaged using the BioImaging system (UVP, Upland, CA, USA). Ubiquitously expressed glyceraldehyde-3-phosphate dehydrogenase (Gapdh) was used as a control. Quantitative real-time PCR for the tested genes was performed on an ABI 7500 (Applied Biosystems, Life Technologies Corporation., Foster, Calfornia, US) system using the TaqMan real-time PCR probe primer mixture according to a previous study by Kagami *et al.* [24].

### DNA methylation analysis via bisulfite modification

Genomic DNA was extracted via proteinase K digestion and phenol/chloroform extraction [25], denatured with NaOH and modified using sodium bisulfite and hydroquinone. Bisulfite-treated DNA was purified using a Wizard DNA clean-up system (Promega, Madison, WI, USA), following the manufacturer’s instructions. DNA was precipitated with ethanol after treatment with NaOH and eluted into 50 μl distilled water. The final products were stored at −80°C until use.

Bisulfite treatment was performed using an EpiTect Bisulfite Kit (Qiagen, Hilden, Germany) according to the manufacturer’s manual. Bisulfite-converted DNA was amplified using nested PCR. Primer sequences are shown in Additional file 1. Each 25 μl PCR reaction mixture contained 4 μl of bisulfite-treated DNA, and reactions were performed according to the method described by Kagami*et al*. [26,27]. The presence of amplified products was confirmed by electrophoresis on a 1.5% agarose gel.

PCR products were retrieved and ligated into the pMD19-T Vector System (TaKaRa). After transformation via heat shock into 200 μl competent Top10 cells (Tiangen, Beijing, China), colonies were isolated and cultured. For each sample, 15 positive clones were sequenced.

### Ascorbic acid supplementation during hESC culture

hpESCs were cultured in knockout (D)MEM medium supplemented with 20% KnockOut Serum Replacement (Gibco, Life Technologies Corporation., Carlsbad, Calfornia, US), 1 mM glutamine (Sigma, Sigma, St . Louis, Missouri, US), 1% non-essential amino acids (Gibco), 0.1 mM 2-mercaptoethanol (Sigma), 50 UI/ml penicillin (Sigma), and 50 UI/ml streptomycin (Sigma) mixture. At passage 21, 100 μg/ml ascorbic acid were added to the ESC culture medium until the end of the experiment. hpESCs cultured without ascorbic acid were taken as the control group.

### Statistical analysis

Data were analyzed using SPSS 17.0 software. The differences between the two groups were calculated with the chi-square test, and those among multiple groups with one-way analysis of variance (ANOVA). Data were considered significantly different at *P* values less than 0.05.

## Results

### Derivation and identification of hpESCs

**Figure 1:**
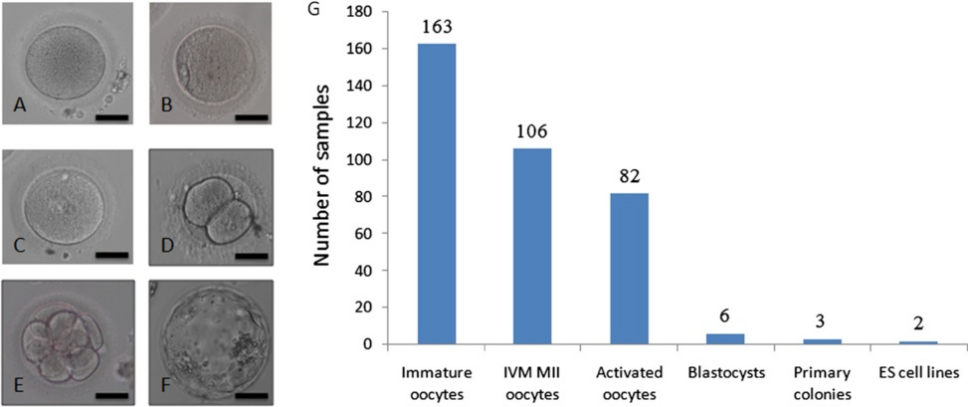
Development of human parthenogenetic embryonic stem cells. **(A)** Immature oocytes; **(B)** Mature metaphase II oocytes; **(C)** Zygote with two pronuclei; **(D)** two-cell embryo; **(E)** eight-cell embryo; **(F)** Blastocyst; **(G)** Development from immature oocytes to human parthenogenetic embryonic stem cells. Bar is 50 μm.

In total, 106 mature oocytes at the MII stage were obtained from 163 immature oocytes (112 GV and 51 MI oocytes). Among these, 82 oocytes were successfully activated, from which six blastocysts were developed. Four of the six blastocysts displayed regular morphology, including trophectoderm cells with a willow leaf shape and compacted inner cell mass. Three primary colonies formed and grew after five days, two of which were propagated until the present time (designated BGHPES-1 and BGHPES-2). Representative oocyte or embryo morphologies and derivation efficiencies are shown in Figure 1.

**Figure 2:**
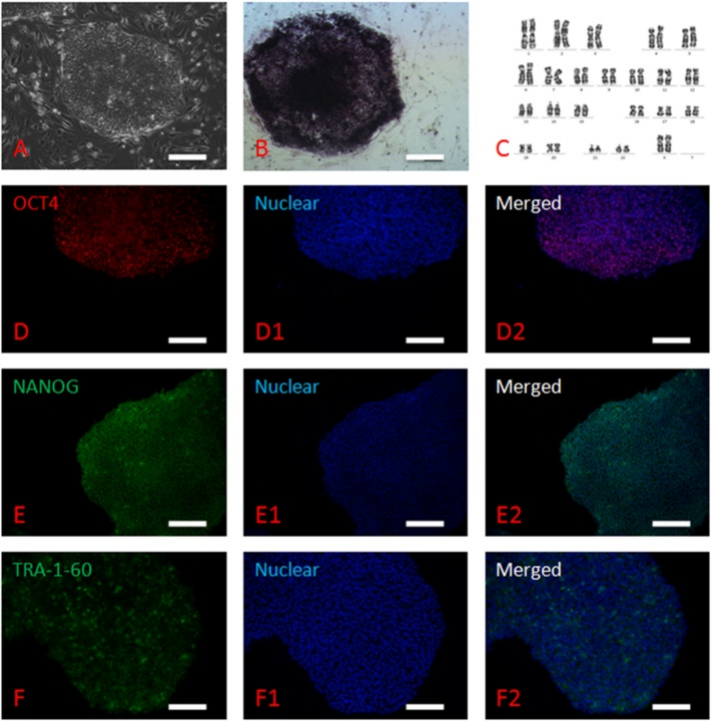
Identification of human parthenogenetic embryonic stem cells. **(A)** Colony of human parthenogenetic embryonic stem cells; **(B)** positive staining for alkaline phosphatase; **(C)** normal 46, XX karyotype at passage 20; **(D)** positive staining for OCT4; **(E)** positive staining for NANOG; **(F)** positive staining for TRA-1-60; (D1-F1) nuclear staining with Hoechst 33342; (D2-F2) merged images for OCT4, NANOG and TRA-1-60. Bar is 100 μm.

**Figure 3:**
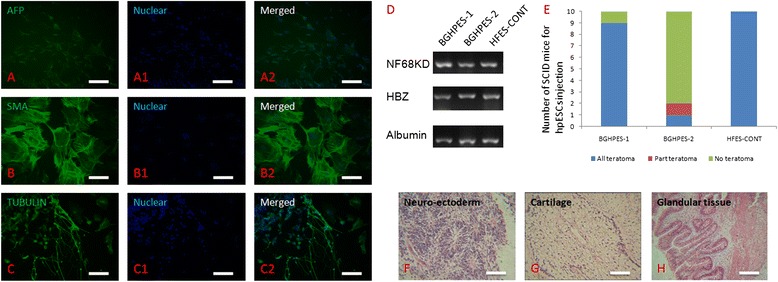
Differentiation abilities of human parthenogenetic embryonic stem cells. *In vitro* differentiated EBs displayed **(A)** positive AFP staining (endoderm), **(B)** positive SMA staining (mesoderm), **(C)** positive TUBULIN staining (ectoderm), and **(D)** expression of genes from endoderm (NF68KD), mesoderm (HBZ) and ectoderm (Albumin). Bar is 50 μm. **(E)** Efficiency of teratoma formation upon injection of human parthenogenetic embryonic stem cells into SCID mice; **(F)** neuro-ectoderm from ectoderm in teratoma; **(G)** cartilage from mesoderm in teratoma; **(H)** glandular tissue from endoderm in teratoma. Bar is 100 μm. EB, embryoid bodies; SCID, severe combined immunodeficiency.

The two hpESCs lines were propagated every four to seven days, and colonies displayed typical hESC morphology, including higher nuclear/cytoplasm ratio and compacted cell conjunction. BGHPES-1 and -2 lines were positive for AP activity and pluripotent markers, including OCT-4 and NANOG, as well as the cell surface marker, TRA-1-60. Moreover, these ESCs displayed the normal chromosome karyotype, 46 XX (Figure 2). Upon differentiation *in vitro*, both ESC lines contributed to derivatives from all three germ layers, as confirmed by both immunofluorescence staining and RT-PCR (Figure 3A-C and D). Following injection into SCID mice, teratomas formed and grew (Figure 3E). However, the differentiation abilities of the two ESC lines were variable. For BGHPES-1, teratoma was observed in nine out of ten non-SCID mice, and derivatives from all three germ layers were identified. In contrast, for BGHPES-2, two teratomas were observed among ten non-SCID mice, but only one contained derivatives from all three germ layers and the other contained derivatives from ectoderm and mesoderm layers only. HFES-CONT lines contributed to teratoma formation with 100% efficiency (Figure 3F).

### Gene expression and methylation modifications in the Dlk1-Dio3 region

**Figure 4:**
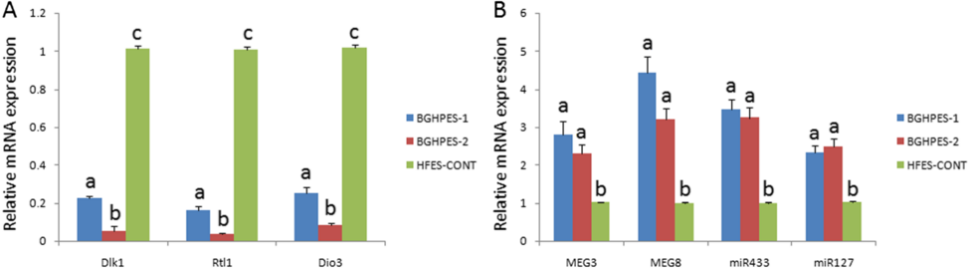
Relative mRNA expression of parental genes in the Dlk1-Dio3 region. **(A)** Expression of paternal genes was significantly higher in human fertilized embryonic stem cells. Dlk1-Dio3 genes in BGHPES-1 were expressed at significantly higher levels, compared to BGHPES-2. **(B)** Maternally expressed genes were significantly up-regulated with no significant differences in both BGHPES-1 and -2, compared with human fertilized embryonic stem cells. Different letters indicate significant differences among data (*P* <0.05) while the same letters imply no differences (*P* >0.05).

Imprinted gene expression profiles in the Dlk1-Dio3 region were determined, including those of paternally expressed genes, Dlk1, Rtl1 and Dio3, and maternally expressed genes, Meg3, Meg8 and antisense Rtl1 (encoding miR433 and miR127). The results appeared consistent with methylation modification data. Decreasing expression of paternal genes was observed, compared with the HFESC-CONT line. However, paternally expressed genes in BGHPES-1 were up-regulated, compared with those of BGHPES-2 (Figure 4A). With regard to maternally expressed genes, two- to four-fold enhanced expression was observed in both BGHPES-1 and -2, compared with normal control. No differences in expression patterns of maternal genes were evident between the two hpESC lines, distinct from data obtained for paternally expressed genes (Figure 4B).

**Figure 5:**
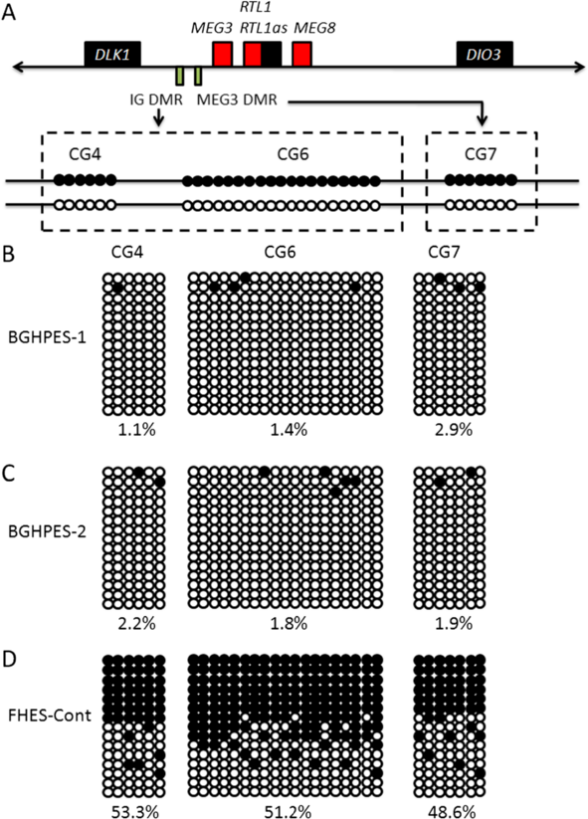
Bisulfite sequencing analysis of IG-DMR (CG4 and CG6) and MEG3-DMR (CG7) using DNA samples from human parthenogenetic and fertilized embryonic stem cells. **(A)** Schematics of IG-DMR and MEG3-DMR in Dlk1-Dio3 imprinting clusters. Normally, CG4 and CG6 structures from IG-DMR and CG7 from MEG3-DMR were methylated (black dots) in paternally derived chromosomes, and unmethylated (white dots) in maternally derived chromosomes; BGHPES-1 **(B)** and -2 **(C)** and control FHES-Cont **(D)** data. Each horizontal line indicates a single subcloned allele. Hypomethylation was observed in BGHPES-1 and -2, and balanced methylation in control cells. IG-DMR, intergenic differentially methylated region.

Germline-derived primary Dlk1-Meg3 intergenic differentially methylated region (IG-DMR) and postfertilization-derived secondary Meg3-DMR were further analyzed. Both DMRs were hypermethylated after paternal transmission and hypomethylated after maternal transmission in the body (Figure 5A). Our results disclosed methylation modifications in approximately 2% of DMRs in BGHPES-1 and -2 (Figure 5B and C). In the HFES-CONT line, half of these regions were methylated, indicating balanced expression of imprinted genes in this cluster (Figure 5D).

To confirm these findings, we ascertained whether the other three hpESCs have been established in Guangzhou before, named GHPES-1, -2 and -3. Among the three hpESC lines, GHPES-1 and -2 contributed to derivatives from all three germ layers in teratoma with high frequency (10/12 and 9/12, respectively), while GHPES-3 failed to form teratoma, even after ESC injection more than ten times (1/12) [See Additional file 2]. Gene expression profiling revealed a significant increase in the levels of paternally expressed genes in GHPES-1 and -2, compared with GHPES-3 [See Additional file 2]. In contrast, no differences were observed in the expression profiles of maternally expressed genes [See Additional file 2]. The proportion of DMR methylation modifications remained at the 1% to 5% level, with no significant differences among the three hpESC lines [See Additional file 3].

### Ascorbic acid supplement alters the levels of paternally expressed genes in the Dlk1-Dio3 region

**Figure 6:**
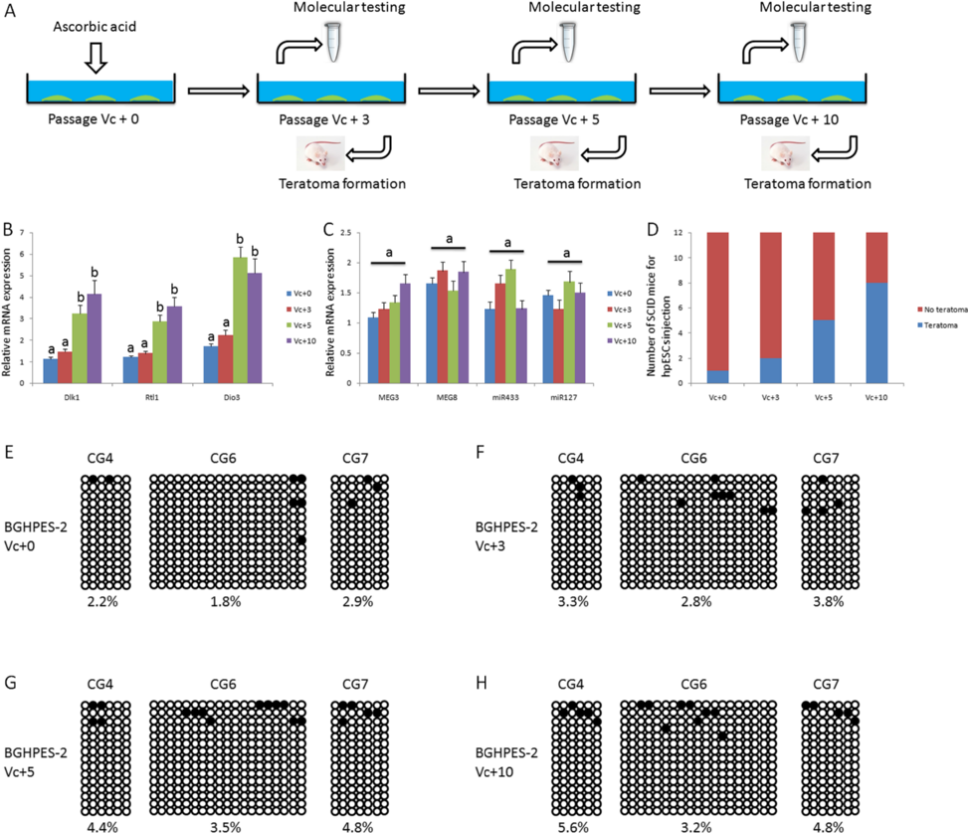
Improvement in pluripotency of BGHPES-2 after treatment with ascorbic acid. **(A)** Procedure of ascorbic acid treatment. At passages 3, 5 and 10 after treatment with ascorbic acid, a portion of cells was collected for molecular testing and another portion injected into SCID mice to form teratomas. **(B)** Paternally expressed genes were up-regulated in BGHPES-2 at passages 5 and 10. **(C)** No differences were observed for maternally expressed genes in BGHPES-2 at passage 0, 3, 5 and 10. **(D)** Rate of teratoma formation was increased in BGHPES-2 at passage 5 and 10. No differences in methylation modifications of IG-DMR and MEG3-DMR in BGHPES-2 were detected at passages 0 **(E)**, 3 **(F)**, 5 **(G)** and 10 **(H)**. Different letters indicate significant differences among data (*P* <0.05) while the same letters are used to signify no differences (*P* >0.05). IG-DMR intergenic differentially methylated region; SCID, severe combined immunodeficiency.

**Figure 7:**
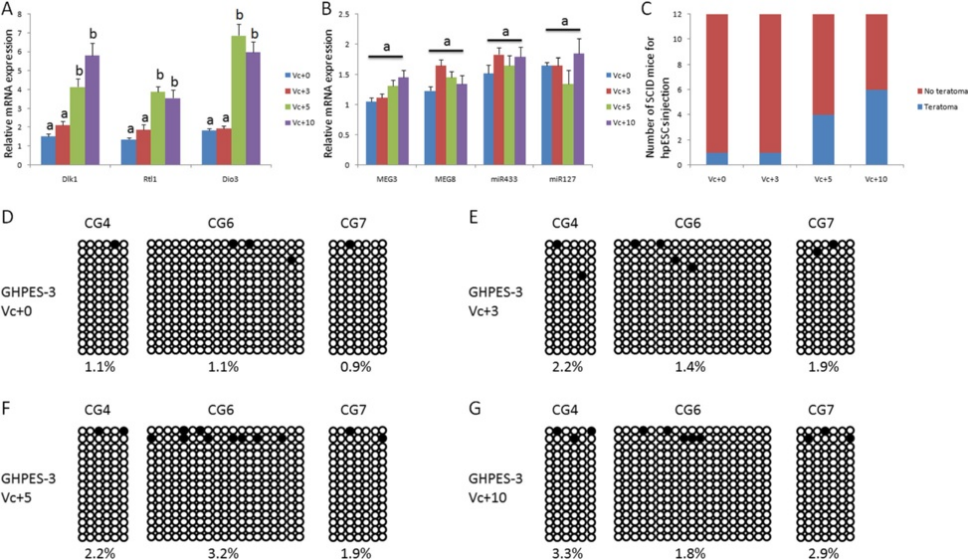
Improvement in pluripotency of GHPES-3 after ascorbic acid treatment. **(A)** Paternally expressed genes were up-regulated in GHPES-3 at passage 5 and 10. **(B)** No differences were observed for maternally expressed genes in GHPES-3 at passages 0, 3, 5 and 10. **(C)** Rate of teratoma formation was increased in GHPES-3 at passages 5 and 10. No differences in methylation modification of IG-DMR and MEG3-DMR in GHPES-3 were detected at passages 0 **(D)**, 3 **(E)**, 5 **(F)** and 10 **(G)**. Different letters indicate significant differences among data (*P* <0.05) while the same letters are used to signify no differences (*P* >0.05). IG-DMR intergenic differentially methylated region.

To establish whether ascorbic acid (also known as Vc) affects the pluripotency of hpESCs, culture media of the five hpESC lines were supplemented with 100 μg/ml compound. After propagating ESCs for 10 passages, relatively higher expression of paternally imprinted genes was detected at passages Vc + 3, Vc + 5 and Vc + 10 in BGHPES-2 and GHPES-3 cell lines. In the BGHPES-2 line, paternal gene expression was increased at passage 5 after Vc treatment, while expression patterns of maternal genes remained unchanged. Upon injection of hpESCs into SCID mice, teratoma formation efficiency was significantly increased with prolongation of Vc treatment duration (Figure 6). Expression patterns of GHPES-3 genes displayed similar changes to those of BGHPES-2 (Figure 7). However, ascorbic acid supplementation did not affect methylation of IG-DMR and MEG3-DMR in both hpESC lines. In the three other hpESCs (BGHPES-1, GHPES-1 and GHPES-2) and control hfESCs, gene expression, teratoma formation and methylation modification remained unaffected by ascorbic acid treatment [See Additional files 4, 5, 6 and 7]. Moreover, we observed that the expression level of Dlk1, Rtl1 and Dio3 in all five hpESCs was still significantly lower than control hfESCs, although Vc supplementation can enhance these gene expression levels in BGHPES-2 and GHPES-3 [See Additional file 8].

To confirm whether differences in paternally expressed genes in Dlk1-Dio3 contribute to the *in vivo* differentiation ability of hpESCs, expression profiles of 25 paternal and 20 maternal genes were further examined. Gene cluster data from paternally expressed genes indicated a close correlation between BGHPES-2 and GHPES-3 that did not form teratomas easily and significantly lower expression of Dlk1, Rtl1 and Dio3 in both lines. However, after ascorbic acid treatment, this correlation between BGHPES-2 and GHPES-3 cell lines was not evident, and the characteristics of paternally expressed genes in the Dlk1-Dio3 cluster normalized [See Additional file 9]. No significant changes in the levels of maternally expressed genes were observed [See Additional file 10].

## Discussion

Data from the present study suggest that the differences in gene expression and methylation modifications in the Dlk1-Dio3 region contribute to the differentiation ability of hpESCs. Ascorbic acid supplementation narrows this gap and enhances the ability of ESCs to form teratomas *in vivo* by promoting paternal imprinted gene expression.

hpESCs are derived in multiple laboratories, and a large proportion form teratomas after injection into SCID mice, which appears similar to findings with hfESCs [7-10]. However, two important facts should be taken into consideration. One is that some established hpESC lines fail to differentiate and form teratomas in SCID mice, and the other is that no studies to date have focused on the efficiency of teratoma formation for pESCs. In the current study, efficiency of teratoma formation was at least 70% in three of our hpESC lines, but ranged from only 8.3% to 20% in the other two lines. Based on the established criteria, these five hpESC lines are categorized as normal ESCs. However, limited differentiation ability restricts the potential clinical application of some of these cells. The differentiation ability of pESCs has been reported previously by Park and co-workers, who showed that EBs differentiated from mouse pESCs display growth retardation and only contribute to derivatives of the endodermal layer [28].

Several methods have been developed to improve the pluripotency of pESCs. Hikichi and co-workers established a new type of pESCs by transferring traditional ESC nuclei into oocytes and deriving these pESCs again at the blastocyst stage (NT-pES), leading to two to five times enhancement of differentiation ability, compared with the original pESCs [29]. The same group reported development of parthenogenetic embryos to term using the serial nuclear transfer method [30]. Epigenetic modification was evidently altered during reprogramming mediated by nuclear transfer, and aberrant methylation observed in cloned embryos and animals. In addition to the nuclear transfer method, embryo aggregation at the eight-cell stage was applied to improve imprinted gene expression in pESCs [31]. Turovets *et al*. treated hpESCs with trichostatin A, a potent histone deacetylase inhibitor, facilitating differentiation into the definitive endoderm lineage [32]. In the present study, treatment of hpESCs with ascorbic acid partly re-activated paternally imprinted gene expression and, consequently, enhanced differentiation potential by promoting teratoma formation.

Ascorbic acid is accepted as a beneficial agent for somatic reprogramming into iPSCs, which acts biologically via Jhdm1a/Jhdm1b to enhance reprogramming efficiency and maintain the imprinting status of the Dlk1-Dio3 region to sustain reprogramming fidelity [33]. Similar to pESCs, iPSCs display impotent germ-line transmission ability via teraploid complementation. Stadtfeld *et al*. treated mouse iPSCs with ascorbic acid at the primary reprogramming stage, and observed increasing efficiency of mouse generation via teraploid complementation [17]. The authors demonstrated that ascorbic acid specifically attenuates hypermethylation of maternal imprinting genes in Dlk1-Dio3 clusters, similar to our results.

Dlk1-Dio3 is an evolutionarily conserved chromosome region located in chromosome 12 in mice and chromosome 14 in humans [34]. Interestingly, Dlk1-Dio3 aberrations induce growth defects in both mice and humans with similar phenotypes, indicating that the biological function of Dlk1-Dio3 is analogous in these two species [35-37]. The Dlk1-Dio3 region appears critical for the pluripotency of iPSCs and tumor promotion [15,38,39]. Earlier data from two groups suggest that hypermethylation of maternally imprinted genes in Dlk1-Dio3 of mouse iPSCs induces abnormal silencing of the locus [17,40], in contrast to our finding that maternally imprinted genes are overexpressed and paternally imprinted genes are silenced in hpESCs. Another study by Henzler and co-workers confirmed that overexpression of Dlk1-Dio3 miRNA early in reprogramming reduces reprogramming efficiency [41], in accordance with our findings. The results indicate that aberrant expression of imprinted genes in this region induces reprogramming deficiency, regardless of higher or lower expression levels.

Re-activation of paternally expressed genes in the Dlk1-Dio3 cluster appears beneficial for regaining pESC pluripotency in mouse. Jiang *et al*. suggested that activation of paternally expressed imprinted genes in pESCs induces germline transmission when injected into blastocysts [19]. Another study by Li and co-workers indicated that the culture environment *in vitro* induces epigenetic changes in paternally expressed imprinted genes, thereby increasing the pluripotency of pESCs [42]. We additionally observed differential expression of specific paternally expressed genes in established hpESCs. However, normal expression of genes in the Dlk1-Dio3 cluster appears critical for pluripotency. Interestingly, Dlk1, Rtl1 and Dio3 gene levels were enhanced upon treatment of hpESCs with ascorbic acid, while IG-DMR and MEG3-DMR methylation patterns remained unchanged, indicating the involvement of other regulatory mechanisms in this modification. Hiura and co-workers demonstrated that valproic acid (VPA) enhances the pluripotency of human iPSCs without methylation modification of DMR [43]. Thus, the DMR methylation process may not be correlated with activation of paternally expressed genes in the Dlk1-Dio3 region, supporting the contribution of other mechanisms in regulation of expression.

Menzorov *et al*. found that mouse ESCs can contribute into teratoma and even in chimera although their Dlk1-Dio3 imprinting control region is hypomethylated [44]. This result is similar to our findings, which confirmed that poor methylation seems not to disturb ESCs pluripotency, but in our results, important paternally expressed genes in this imprinting region were up-regulated and positively related to ESCs differentiation ability, which indicated that methylation is not the only regulation mode for these genes in the Dlk1-Dio3 region. Non-coding RNAs may be an undelying regulator for the expression of these genes. In the Dlk1-Dio3 region, the maternally expressed genes can produce non-coding RNAs, including mir-431, mir-433, mir-127, mir-432 and mir-136. Liu *et al*. suggested that microRNAs in the Dlk1-Dio3 region can potentially target components of the polycomb repressive complex 2 (PRC2) and may form a feedback regulatory loop resulting in the expression of all genes and non-coding RNAs encoded by this region [15]. Regarding the fact that microRNA expression can be regulated by ascorbic acid treatment [45], it is possible that ascorbic acid indirectly changed the expression level of paternal genes by microRNA regulation.

Imprinting genes have shown a tremendous potential to regulate their pluripotency and multi-differentiation ability. During cell differentiation and tissue formation, it is critical that genomic demethylation and *de novo* methylation take place for lineage specification and to establish tissue-specific methylation patterns. Often, non-fertilized-originated human pluripotent stem cells showed abnormal epigenetic modification and imprinting gene expression, including nuclear transfer embryonic stem cells [46] and iPSCs [47]; however, hpESCs have a stable epigenetic modification mode, which is different from the dynamics and inconsistent epigenetic status in the other two kinds of pluripotent stem cells. In 2004, Kono *et al*. proved first that modification of H19 and IGF2 in oocytes can result in the birth of parthenogenetic mice [48]. This indicated that H19-IGF2 was important to parthenogenesis, which suggested that manipulation of imprinting genes can reverse parthenote to fertilization-like status. In Mitalipov's study, he suggested that hypermethylation within the IGF2/H19 IC in all analyzed primate ESC lines resulted in a risk of cellular overproliferation and tumor formation [49]. Recently, Yin *et al*. demonstrated that knockdown of H19 can improve mouse pESC differentiation potential in the lineages of ectoderm and mesoderm [50]. In further study, H19-Ifg2 and Dlk1-Dio3 imprinting region co-modification may have the greatest impact on improvement of the differentiation ability of pESCs.

## Conclusions

In conclusion, the efficiency of teratoma formation by hpESCs is improved with ascorbic acid treatment, leading to enhancement of potential differentiation ability. Ascorbic acid has beneficial effects on general human physiology and may, therefore, be safely used as a stimulatory drug to facilitate clinical application of hpESCs.

## Additional files

Additional file 1: Table S1. Gene primer sequence for RT-PCR. Additional file 2: Figure S1. Teratoma formation and relative mRNA expression of parental genes in the Dlk1-Dio3 region in GHPES-1, -2 and -3 groups. (A) Rate of teratoma formation was decreased in GHPES-3; (B) Expression of paternal genes was significantly higher in human fertilized embryonic stem cells. Dlk1-Dio3 genes in GHPES-1 and -2 were expressed at significantly higher levels, compared to GHPES-3; (C) Maternally expressed genes were significantly up-regulated with no significant differences in among GHPES-1, -2 and -3, compared with human fertilized embryonic stem cells. Different letters indicate significant differences among data (*P* <0.05) while the same letters imply no differences (*P* >0.05). Additional file 3: Figure S2. Bisulfite sequencing analysis of IG-DMR (CG4 and CG6) and MEG3-DMR (CG7) using DNA samples from human parthenogenetic and fertilized embryonic stem cells. (A) GHPES-1, -2 and -3 data. Each horizontal line indicates a single subcloned allele. Hypomethylation was observed in BGHPES-1, -2 and -3. Additional file 4: Figure S3. Improvement in pluripotency of BGHPES-1 after treatment with ascorbic acid. (A) Paternally expressed genes were not changed in BGHPES-1 at each passage. (B) No differences were observed for maternally expressed genes in BGHPES-1 at passages 0, 3, 5 and 10. (C) Rate of teratoma formation was increased in BGHPES-1 at passage 5 and 10; no differences in methylation modifications of IG-DMR and MEG3-DMR in BGHPES-1 were detected at passages 0 (D), 3 (E), 5 (F) and 10 (G). Different letters indicate significant differences among data (*P* <0.05) while the same letters are used to signify no differences (*P* >0.05). Additional file 5: Figure S4. Improvement in pluripotency of GHPES-1 after treatment with ascorbic acid. (A) Paternally expressed genes were not changed in GHPES-1 at each passage. (B) No differences were observed for maternally expressed genes in GHPES-1 at passages 0, 3, 5 and 10. (C) Rate of teratoma formation was increased in GHPES-1 at passages 5 and 10; no differences in methylation modifications of IG-DMR and MEG3-DMR in GHPES-1 were detected at passages 0 (D), 3 (E), 5 (F) and 10 (G). Different letters indicate significant differences among data (*P* <0.05) while the same letters are used to signify no differences (*P* >0.05). Additional file 6: Figure S5. Improvement in pluripotency of GHPES-2 after treatment with ascorbic acid. (A) Paternally expressed genes were not changed in GHPES-2 at each passage. (B) No differences were observed for maternally expressed genes in GHPES-2 at passages 0, 3, 5 and 10. (C) Rate of teratoma formation was increased in GHPES-2 at passage 5 and 10; no differences in methylation modifications of IG-DMR and MEG3-DMR in GHPES-2 were detected at passages 0 (D), 3 (E), 5 (F) and 10 (G). Different letters indicate significant differences among data (*P* <0.05) while the same letters are used to signify no differences (*P* >0.05). Additional file 7: Figure S6. Improvement in pluripotency of HFES-CONT after treatment with ascorbic acid. (A) Paternally expressed genes were not changed in HFES-CONT at each passage. (B) No differences were observed for maternally expressed genes in HFES-CONT at passages 0, 3, 5 and 10. (C) Rate of teratoma formation was increased in HFES-CONT at passage 5 and 10; no differences in methylation modifications of IG-DMR and MEG3-DMR in HFES-CONT were detected at passages 0 (D), 3 (E), 5 (F) and 10 (G). Different letters indicate significant differences among data (*P* <0.05) while the same letters are used to signify no differences (*P* >0.05). Additional file 8: Figure S7. Dynamic changes of paternally expressed genes in hpESCs after ascorbic acid treatment. In Vc + 0 (A) and Vc + 3 (B) groups, the expression level of Dlk1, Rtl1 and Dio3 was significantly lower in BGHPES-2 and GHPES-3, compared with BGHPES-1, GHPES-1 and GHPES-2; however, the expression level of these three genes in all five hpESCs lines was significantly lower than that in HFES-CONT. In Vc + 5 (A) and Vc + 10 (B) groups, the expression level of Dlk1, Rtl1 and Dio3 in BGHPES-2 and GHPES-3 was enhanced and comparable with BGHPES-1, GHPES-1 and GHPES-2; however, the expression level of these three genes in all five hpESCs lines was still significantly lower than that in HFES-CONT. Different letters indicate significant differences among data (*P* <0.05) while the same letters are used to signify no differences (*P* >0.05). Additional file 9: Figure S8. Cluster analysis of paternally expressed genes in human parthenogenetic embryonic stem cells. (A) Differences in expression of Dlk1, Rtl1 and Dio3 between human parthenogenetic embryonic stem cells that form teratoma and those that do not form teratoma were observed. (B) No regular changes in Dlk1, Rtl1 and Dio3 were evident in human parthenogenetic embryonic stem cells after ascorbic acid treatment. Additional file 10: Figure S9. Cluster analysis of maternally expressed genes in human parthenogenetic embryonic stem cells. No regular changes in 20 maternally expressed imprinting genes were evident in human parthenogenetic embryonic stem cells before (A) or after (B) ascorbic acid treatment.

## Abbreviations

6-DMAP: 6-dimethylaminopyridine
BSA: bovine serum albumin
(D)MEM: (Dulbecco’s) modified Eagle’s medium
DMR: differentially methylated region
EBs: embryoid bodies
hESCs: human embryonic stem cells
hfESCs: human fertilized embryonic stem cells
hpESCs: human parthenogenetic embryonic stem cells
ICSI: intra-cytoplasmic sperm injection
IG-DMR: intergenic differentially methylated region
iPSC: induced pluripotent stem cell
MEF: murine embryonic fibroblasts
PBS: phosphate-buffered saline
pESCs: parthenogenetic ESCs
RT-PCR: real time polymerase chain reaction
SCID: severe combined immunodeficiency
VPA: valproic acid
