# Erratum to: Ascorbic acid improves pluripotency of human parthenogenetic embryonic stem cells through modifying imprinted gene expression in the Dlk1-Dio3 region

**DOI:** 10.1186/s13287-015-0161-7

**Published:** 2015-09-17

**Authors:** Yang Yu, Qian Gao, Hong-cui Zhao, Rong Li, Jiang-man Gao, Ting Ding, Si-yu Bao, Yue Zhao, Xiao-fang Sun, Yong Fan, Jie Qiao

**Affiliations:** Department of Obstetrics and Gynecology, Center of Reproductive Medicine, Peking University Third Hospital, No. 49 HuaYuan North Road, HaiDian District, Beijing, 100191 People’s Republic of China; Key Laboratory of Assisted Reproduction, Ministry of Education, Beijing, 100191 China; Beijing Key Laboratory of Reproductive Endocrinology and Assisted Reproductive Technology, Beijing, 100191 China; Key Laboratory for Major Obstetric Diseases of Guangdong Province, the Third Affiliated Hospital of Guangzhou Medical University, No. 63, Liwan District, Guangzhou City, 510150 Guangdong Province People’s Republic of China

## Erratum

Following the publication of our article [1], we noticed that some incorrect images had been incorporated into figure twoB (included here as Fig. 1b) and threeF-H (included here as Fig. 2f-h) in error. The corrected figures are given below. This correction does not change the results or conclusion of the original study.

**Fig. 1:**
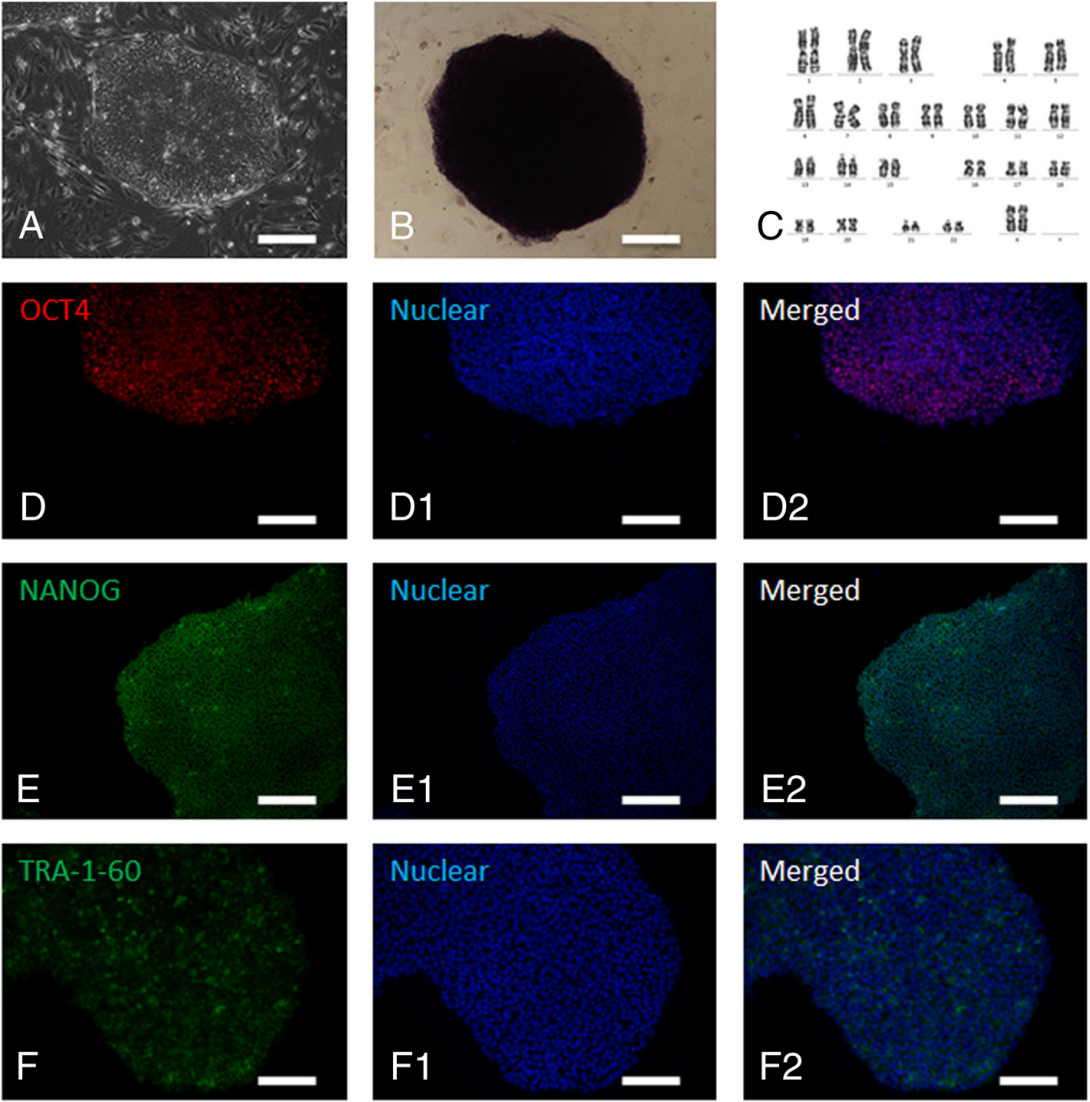
Identification of human parthenogenetic embryonic stem cells. **a** Colony of human parthenogenetic embryonic stem cells; (**b**) positive staining for alkaline phosphatase; **(c)** normal 46, XX karyotype at passage 20; (**d**) positive staining for OCT4; (**e**) positive staining for NANOG; (**f**) positive staining for TRA-1-60; (D1-F1) nuclear staining with Hoechst 33342; (D2-F2) merged images for OCT4, NANOG and TRA-1-60. Bar is 100 μm

**Fig. 2:**
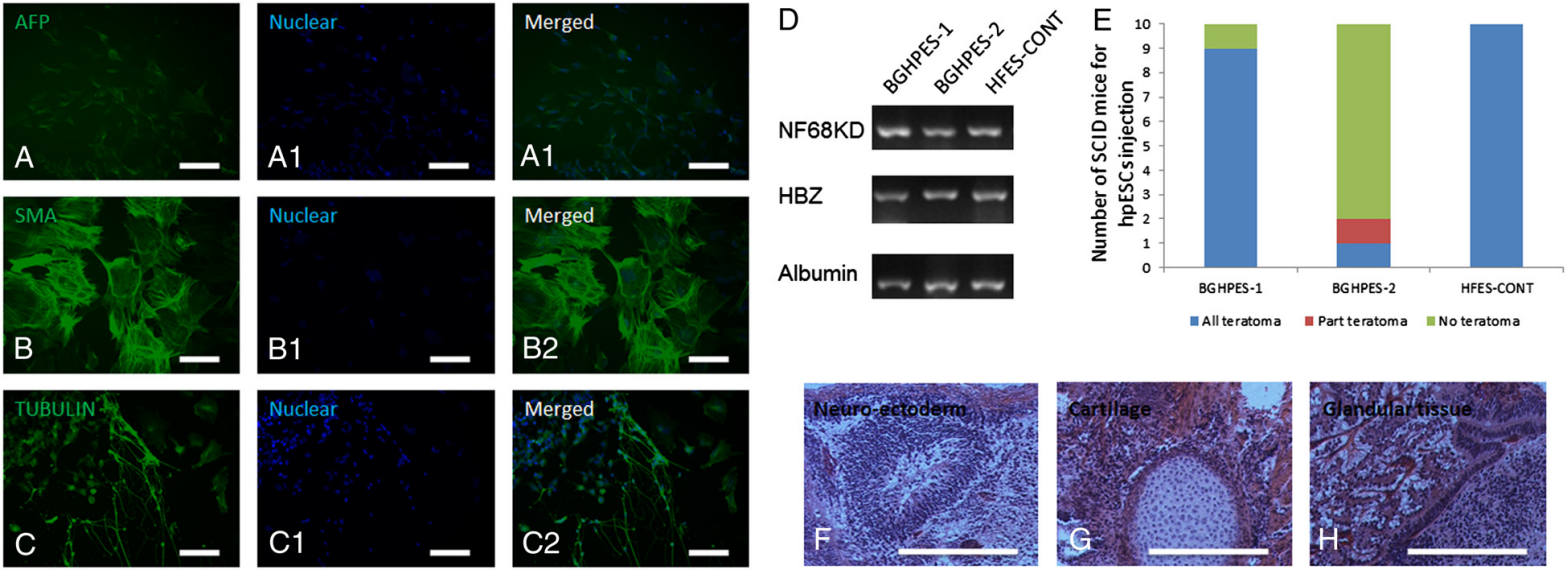
Differentiation abilities of human parthenogenetic embryonic stem cells. *In vitro* differentiated EBs displayed (**a**) positive AFP staining (endoderm), (**b**) positive SMA staining (mesoderm), (**c**) positive TUBULIN staining (ectoderm), and (**d**) expression of genes from endoderm (NF68KD), mesoderm (HBZ) and ectoderm (Albumin). Bar is 50 μm. (**e**) Efficiency of teratoma formation upon injection of human parthenogenetic embryonic stem cells into SCID mice; (**f**) neuro-ectoderm from ectoderm in teratoma; (**g**) cartilage from mesoderm in teratoma; (**h**) glandular tissue from endoderm in teratoma. Bar is 100 μm. EB, embryoid bodies; SCID, severe combined immunodeficiency
