# Supplementary material for: Ascorbic acid improves pluripotency of human parthenogenetic embryonic stem cells through modifying imprinted gene expression in the Dlk1-Dio3 region
Source: Stem Cell Res Ther. 2015 Apr 14;6(1):69. doi: 10.1186/s13287-015-0054-9 (PMC4425892; doi:10.1186/s13287-015-0054-9)
Supplement: Additional file 1: Table S1. — Gene primer sequence for RT-PCR. [file 13287_2015_54_MOESM1_ESM.docx]

Table S1 Primer sets used for PCR reaction

| Gene | Primer Sets |
| --- | --- |
| *NF68KD* | Forward: 5′-TCCCCTGAAGAAATTGGTTAAAAT-3′  Reverse: 5′-GAGTGAAATGGCACGATACCTA-3′ |
| *HBZ* | Forward: 5′- CTGACCAAGACTGAGAGGAC -3′  Rev: 5′- ATGTCGTCGATGCTCTTCAC -3′ |
| *Albumin* | Forward: 5′-TGCTTGAATGTGCTGATGACAGGG-3′  Reverse: 5′-AAGGCAAGTCAGCAGCCATCTCAT-3′ |
| CG4 | Forward: 5′-TTTTATTATTGAATTGGGTTTGTTAGT-3′  Reverse: 5′-ACAATTCCTACTACAAAATTTCAACA-3′ |
| CG6 | Forward: 5′-GTTAAGAGTTTGTGGATTTGTGAGAAATG-3′  Reverse: 5′-CTAAAAATCACCAAAACCCATAAAATCAC-3′ |
| CG7 | Forward: 5′-TTGTGTTTGAATTTATTTTGTTT-3′  Reverse: 5′-CCCCAAATTCTATAACAAATTACT-3′ |
| **LRRTM1** | Forward: 5′-CGAGCAGCAAAGTGAGACAT-3′  Reverse: 5′-GAGTGACAACCTCCATTCAGC-3′ |
| **NAP1L5** | Forward: 5′-CCTACTCGCCAAGATCCAAG-3′  Reverse: 5′-CCTCGTCATCCTCGTACTCC-3′ |
| HYMAI | Forward: 5′-TGAAAGCCACTGCCCTAGAT-3′  Reverse: 5′-TTCTCCTTCAGGTAGGCATGA-3′ |
| PLAGL1 | Forward: 5′-CATTCCAACTGAAGGCTGCT-3′  Reverse: 5′-CTGAGGGTGAGGCTATGGAC-3′ |
| SGCE | Forward: 5′-TGTCAACGCTTCCTGTGTTC-3′  Reverse: 5′-TCTGATGTGGCAAGTTCTGC-3′ |
| PEG10 | Forward: 5′-GCCATAAGAGTGCGTGTGTC-3′  Reverse: 5′-GTTGTTCTCCTCCGACTGCT-3′ |
| MEST | Forward: 5′-TGGGCTTCATCAACTCCTTC-3′  Reverse: 5′-GGGCATTTCTTCCTAAGTGG-3′ |
| COPG2IT1 | Forward: 5′-GGGTTCAGCATTTCACTTCC-3′  Reverse: 5′-CCAGTAGCCATCCCTCCAT-3′ |
| DLGAP2 | Forward: 5′-GGAAGATGAGAAGCGACACG-3′  Reverse: 5′-GAAGGATGAGCCGAACTGAA-3′ |
| IGF2 | Forward: 5′-GACACCCTCCAGTTCGTCTG-3′  Reverse: 5′-CGGAAACAGCACTCCTCAAC-3′ |
| INS | Forward: 5′-GCATCTGCTCCCTCTACCAG-3′  Reverse: 5′-GGTTCAAGGGCTTTATTCCA-3′ |
| KCNQ1OT1 | Forward: 5′-AGAGCGGTGTGGTGACAGA-3′  Reverse: 5′-TTAGGGCTGGCTTTCCAAC-3′ |
| DLK1 | Forward: 5′-CCGAGAAGATCGACATGACC-3′  Reverse: 5′-CTCTGCGGAACTCCAAGAAT-3′ |
| RTL1 | Forward: 5′-CCACTCTCCCTACTGCCTGA-3′  Reverse: 5′-CATCTGCTTCCTTCGGGTTA-3′ |
| DIO3 | Forward: 5′-CCCACTGCTGATGACGAAC-3′  Reverse: 5′-TTACCTCACGGAAACCCAGA-3′ |
| MKRN3 | Forward: 5′-TGCCATTGAGTTTGTTCCAG-3′  Reverse: 5′-ACAGCCATCTGCTTCCTCTC-3′ |
| MAGEL2 | Forward: 5′-ATCTTTGCTGCCACCTTCTG-3′  Reverse: 5′-GCTGGGAAGACACTTGAGGA-3′ |
| NDN | Forward: 5′-AAGAAGTGGTGCAGGAGCAT-3′  Reverse: 5′-GACCAGCGCAAACTCCAT-3′ |
| C15ORF2 | Forward: 5′-ATCCTGCCTATCCCTCCACT-3′  Reverse: 5′-AATGGGAACAAGGTCAGCAG-3′ |
| SNRPN | Forward: 5′-TTTGGGTCTGGTGTTGCTG-3′  Reverse: 5′-CCTCTTCCCTGTGGAGTCAT-3′ |
| PEG3 | Forward: 5′-ACCCTGTCATTCAAGGCTCA-3′  Reverse: 5′-TGCTGTGGACTTTCTGATGG-3′ |
| NNAT | Forward: 5′-AGTTGAGGAAGGAGGGTGGT-3′  Reverse: 5′-GAGGCAGTGAGGAGGAAAGG-3′ |
| L3MBTL | Forward: 5′-ACTTCTGGGTCAATGCCAAC-3′  Reverse: 5′-GCTGAACTCCTCCTCCTTGT-3′ |
| TP73 | Forward: 5′-CACCTCCCAAGGGTTACAGA-3′  Reverse: 5′-CCAGATGGTCATGCGGTACT-3′ |
| PRIM2 | Forward: 5′-CTGGGATTACGGTTGTGAGC-3′  Reverse: 5′-CATGCCTGTAGATGGATTGG-3′ |
| CALCR | Forward: 5′-TGCGGTGGTATTATCTCTTGG-3′  Reverse: 5′-CTCAGCCAGCAGTTGTCATT-3′ |
| TFPI2 | Forward: 5′-CGGATTGAGAACAGGTTTCC-3′  Reverse: 5′-CATTCCCTCCACAGCCAGTA-3′ |
| PPP1R9A | Forward: 5′-GTTAATCCCATCAGCCGAGA-3′  Reverse: 5′-TGATCGTCCCAACAGGTGTA-3′ |
| CPA4 | Forward: 5′-GGCAAGAACGGAGCAGTAAT-3′  Reverse: 5′-GCCAGGTCAGGAAAGTCTG-3′ |
| KLF14 | Forward: 5′-CTCTGGTGGGTTCTCTGGA-3′  Reverse: 5′-TGAGGTGCGACGACTTGTAA-3′ |
| KCNK9 | Forward: 5′-CTCCTTCCTTCCCAGCAGA-3′  Reverse: 5′-CTCCGTGGTCTTGGTCTCAC-3′ |
| H19 | Forward: 5′-TTCAAAGCCTCCACGACTCT-3′  Reverse: 5′-GCTCACACTCACGCACACTC-3′ |
| KCNQ1 | Forward: 5′-GGAAGCCCTCACTGTTCATC-3′  Reverse: 5′-GCTGCGTCACCTTGTCTTCT-3′ |
| CDKN1C | Forward: 5′-GACGCAGAAGAGTCCACCAC-3′  Reverse: 5′-AGTCGTAATCCCAGCGGTTC-3′ |
| SLC22A18 | Forward: 5′-TTCTCCATCGTGCCATACCT-3′  Reverse: 5′-TCTGCGAACCTGCCAAATAC-3′ |
| PHLDA2 | Forward: 5′-TCCAGCTATGGAAGAAGAAGC-3′  Reverse: 5′-CTTGAGGATGGAGTGGAAGC-3′ |
| OSBPL5 | Forward: 5′-AAGGGAATCAAGAAGCCGTA-3′  Reverse: 5′-TGGGACACCTGCTCTGCTA-3′ |
| UBE3A | Forward: 5′-TGCCATTGTTGCTGCTTC-3′  Reverse: 5′-TGGGCTCTTCATCATCTTCT-3′T |
| ATP10A | Forward: 5′-TGCTGGAGAAGGTGAGTGAG-3′  Reverse: 5′-GATGGTGAGTGCGATGAAGA-3′ |
| ZNF597 | Forward: 5′-GATGGGAGAGGAAGGCAAG-3′  Reverse: 5′-TTTCTGGGTAAGGGACAAGG-3′ |
| TCEB3C | Forward: 5′-GAAATCGCCTCCTGTCCA-3′  Reverse: 5′-GAGAGTGCTTCTGGGTTTGC-3′ |
| NLRP2 | Forward: 5′-TCTCCAAAGGCTGCTACTCC-3′  Reverse: 5′-CCGTCCCTATCCTCTTCCTC-3′ |
| GNAS | Forward: 5′-TTTGAGACCAAGTTCCAGGTG-3′  Reverse: 5′-CCACGAAGATGATGGCAGT-3′ |
