# Supplementary figures and images for: Ascorbic acid improves pluripotency of human parthenogenetic embryonic stem cells through modifying imprinted gene expression in the Dlk1-Dio3 region
Source: Stem Cell Res Ther. 2015 Apr 14;6(1):69. doi: 10.1186/s13287-015-0054-9 (PMC4425892; doi:10.1186/s13287-015-0054-9)

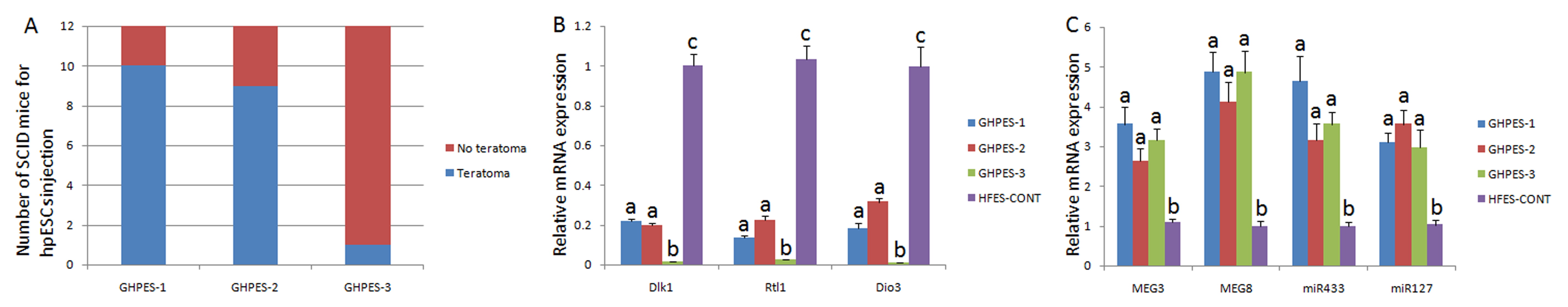

Supplement: Additional file 2: Figure S1. — Teratoma formation and relative mRNA expression of parental genes in the Dlk1-Dio3 region in GHPES-1, -2 and -3 groups. (A) Rate of teratoma formation was decreased in GHPES-3; (B) Expression of paternal genes was significantly higher in human fertilized embryonic stem cells. Dlk1-Dio3 genes in GHPES-1 and -2 were expressed at significantly higher levels, compared to GHPES-3; (C) Maternally expressed genes were significantly up-regulated with no significant differences in among GHPES-1, -2 and -3, compared with human fertilized embryonic stem cells. Different letters indicate significant differences among data (P <0.05) while the same letters imply no differences (P >0.05). [file 13287_2015_54_MOESM2_ESM.tiff]

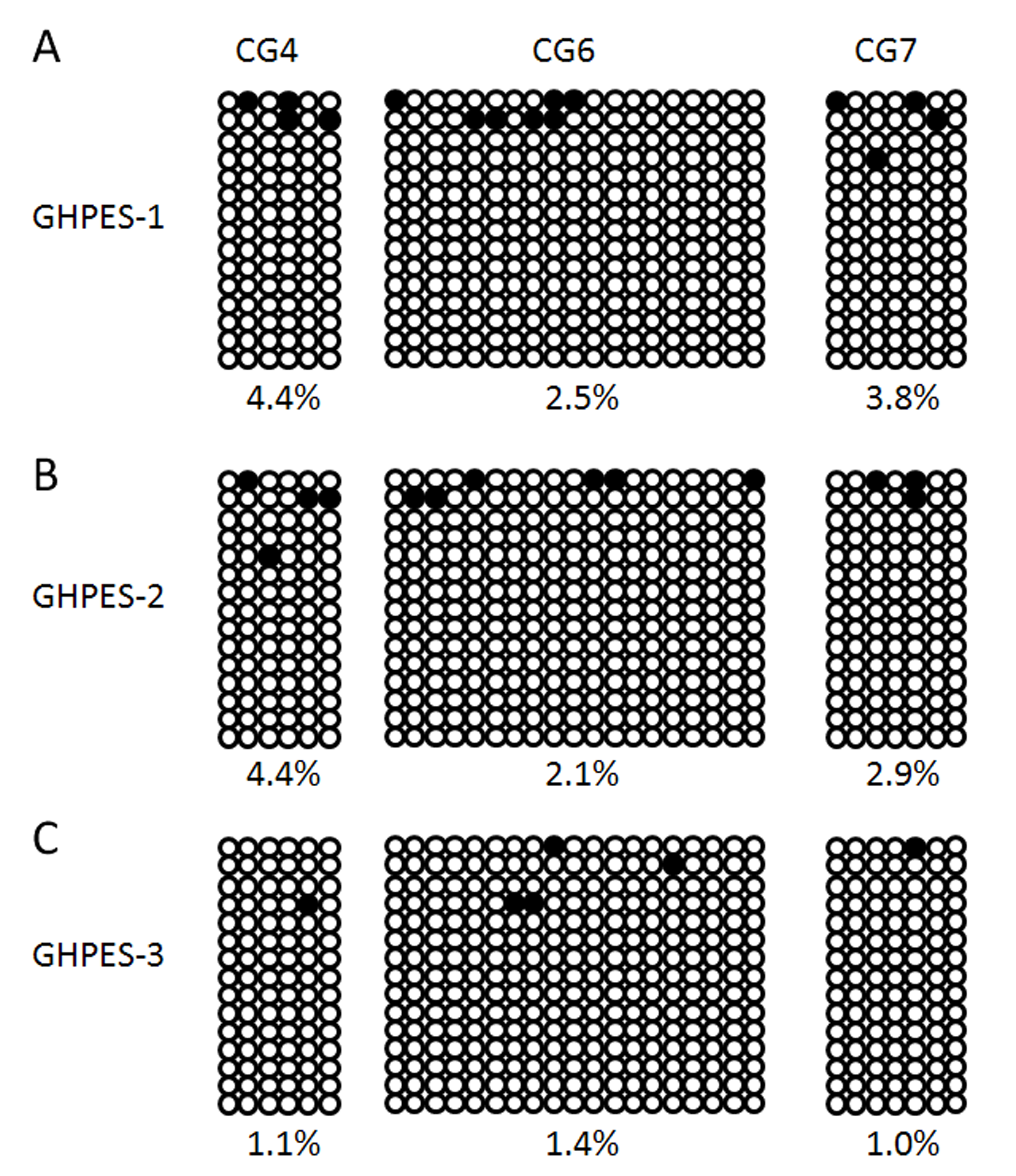

Supplement: Additional file 3: Figure S2. — Bisulfite sequencing analysis of IG-DMR (CG4 and CG6) and MEG3-DMR (CG7) using DNA samples from human parthenogenetic and fertilized embryonic stem cells. (A) GHPES-1, -2 and -3 data. Each horizontal line indicates a single subcloned allele. Hypomethylation was observed in BGHPES-1, -2 and -3. [file 13287_2015_54_MOESM3_ESM.tiff]

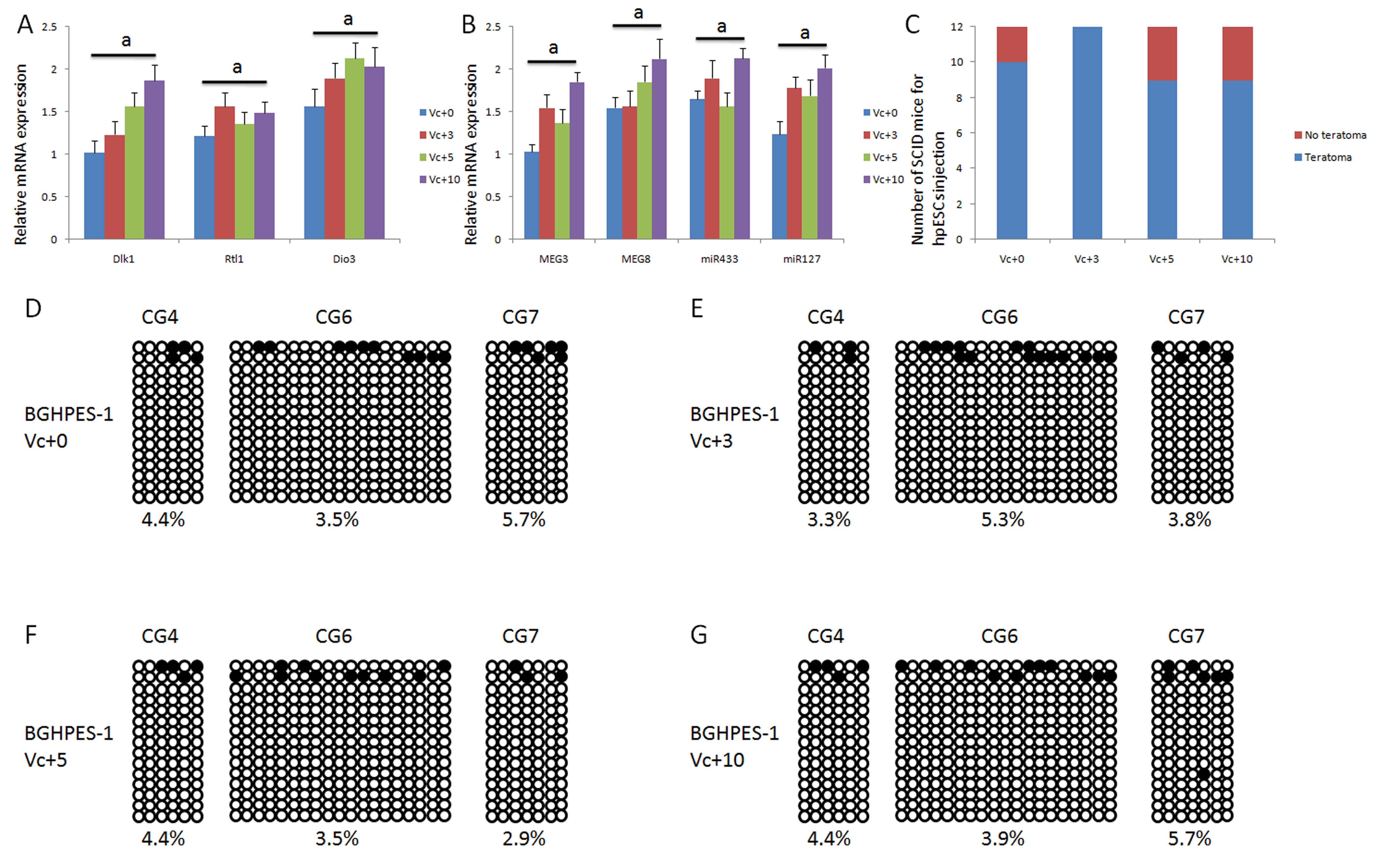

Supplement: Additional file 4: Figure S3. — Improvement in pluripotency of BGHPES-1 after treatment with ascorbic acid. (A) Paternally expressed genes were not changed in BGHPES-1 at each passage. (B) No differences were observed for maternally expressed genes in BGHPES-1 at passages 0, 3, 5 and 10. (C) Rate of teratoma formation was increased in BGHPES-1 at passage 5 and 10; no differences in methylation modifications of IG-DMR and MEG3-DMR in BGHPES-1 were detected at passages 0 (D), 3 (E), 5 (F) and 10 (G). Different letters indicate significant differences among data (P <0.05) while the same letters are used to signify no differences (P >0.05). [file 13287_2015_54_MOESM4_ESM.tiff]

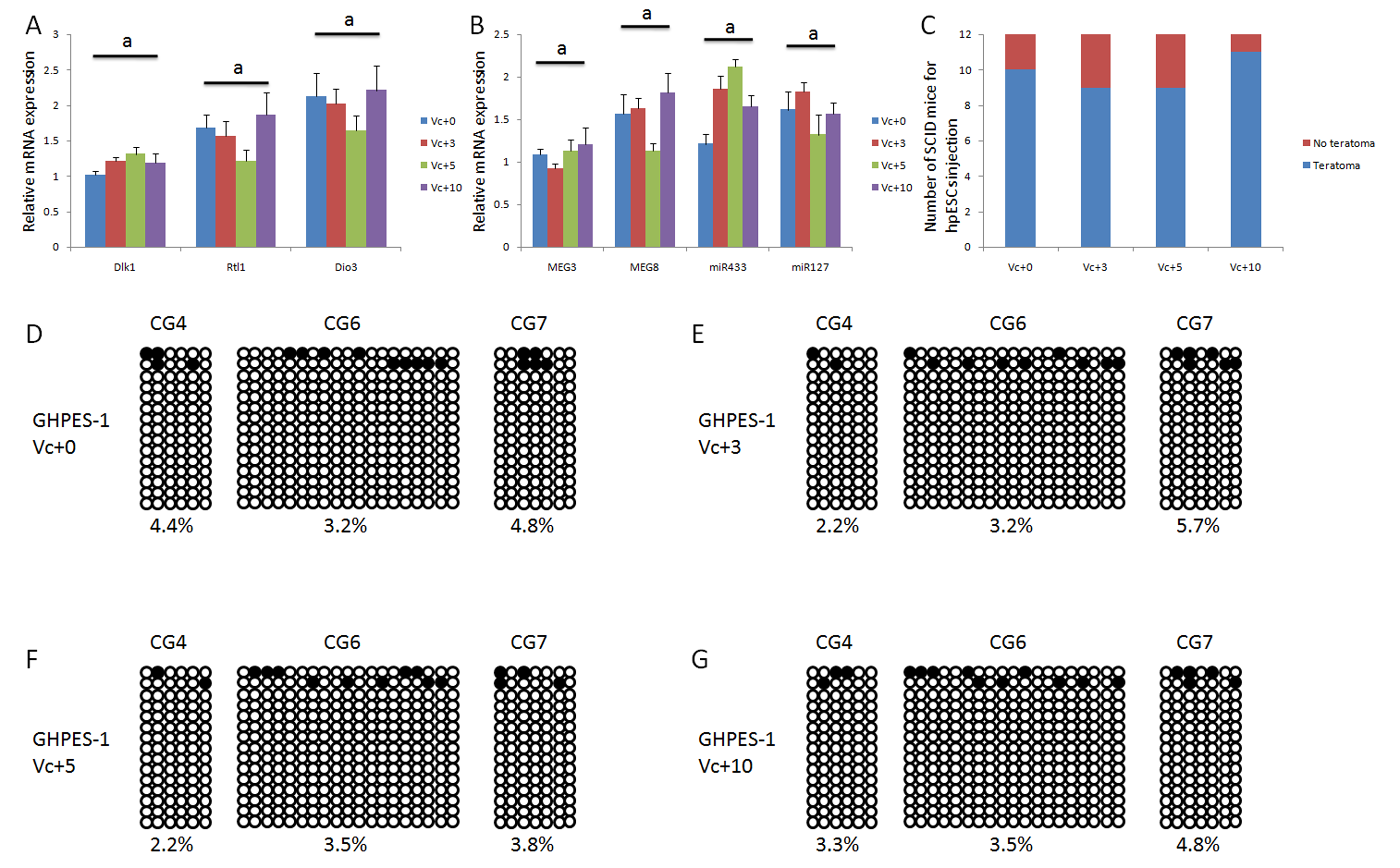

Supplement: Additional file 5: Figure S4. — Improvement in pluripotency of GHPES-1 after treatment with ascorbic acid. (A) Paternally expressed genes were not changed in GHPES-1 at each passage. (B) No differences were observed for maternally expressed genes in GHPES-1 at passages 0, 3, 5 and 10. (C) Rate of teratoma formation was increased in GHPES-1 at passages 5 and 10; no differences in methylation modifications of IG-DMR and MEG3-DMR in GHPES-1 were detected at passages 0 (D), 3 (E), 5 (F) and 10 (G). Different letters indicate significant differences among data (P <0.05) while the same letters are used to signify no differences (P >0.05). [file 13287_2015_54_MOESM5_ESM.tiff]

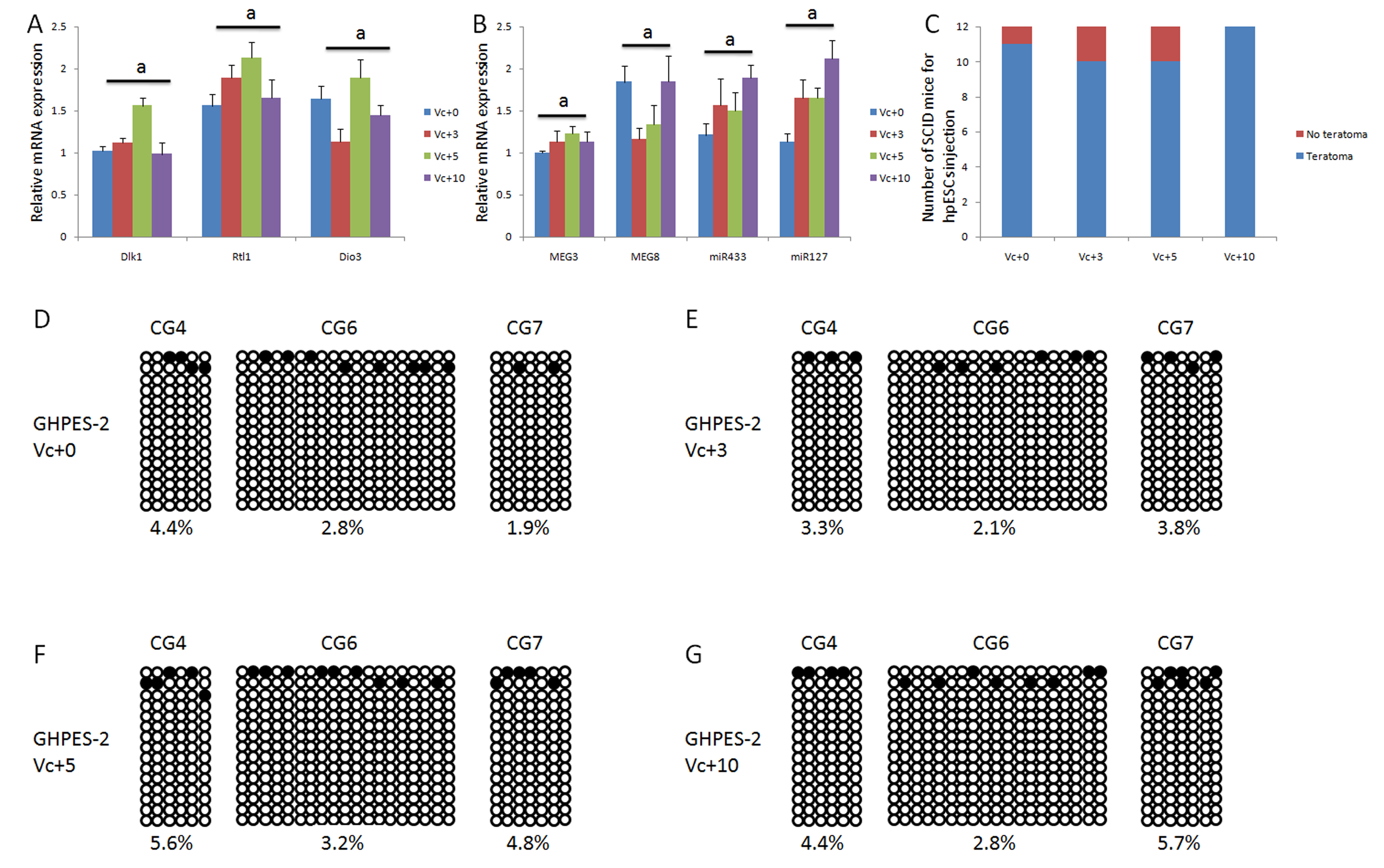

Supplement: Additional file 6: Figure S5. — Improvement in pluripotency of GHPES-2 after treatment with ascorbic acid. (A) Paternally expressed genes were not changed in GHPES-2 at each passage. (B) No differences were observed for maternally expressed genes in GHPES-2 at passages 0, 3, 5 and 10. (C) Rate of teratoma formation was increased in GHPES-2 at passage 5 and 10; no differences in methylation modifications of IG-DMR and MEG3-DMR in GHPES-2 were detected at passages 0 (D), 3 (E), 5 (F) and 10 (G). Different letters indicate significant differences among data (P <0.05) while the same letters are used to signify no differences (P >0.05). [file 13287_2015_54_MOESM6_ESM.tiff]

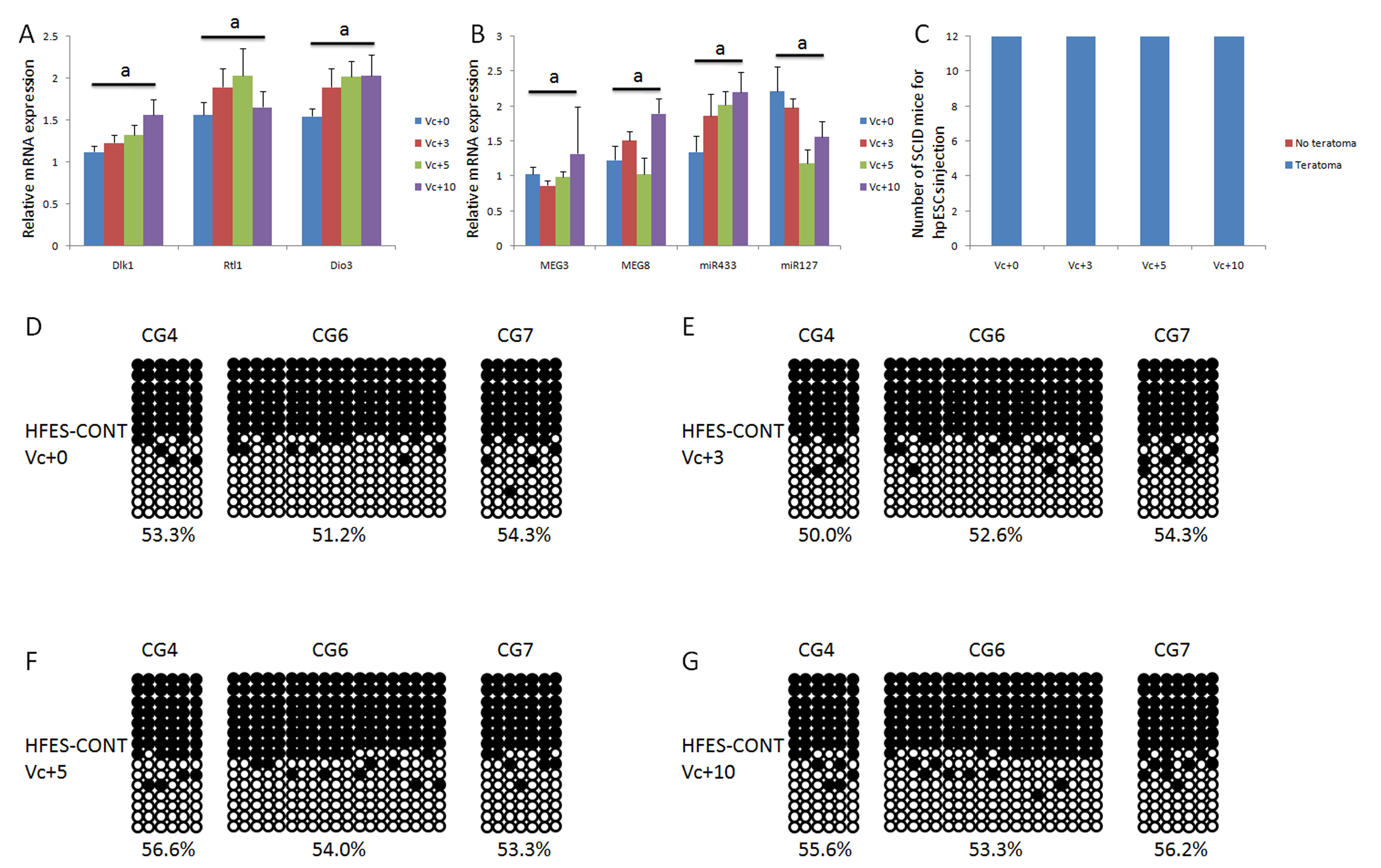

Supplement: Additional file 7: Figure S6. — Improvement in pluripotency of HFES-CONT after treatment with ascorbic acid. (A) Paternally expressed genes were not changed in HFES-CONT at each passage. (B) No differences were observed for maternally expressed genes in HFES-CONT at passages 0, 3, 5 and 10. (C) Rate of teratoma formation was increased in HFES-CONT at passage 5 and 10; no differences in methylation modifications of IG-DMR and MEG3-DMR in HFES-CONT were detected at passages 0 (D), 3 (E), 5 (F) and 10 (G). Different letters indicate significant differences among data (P <0.05) while the same letters are used to signify no differences (P >0.05). [file 13287_2015_54_MOESM7_ESM.tiff]

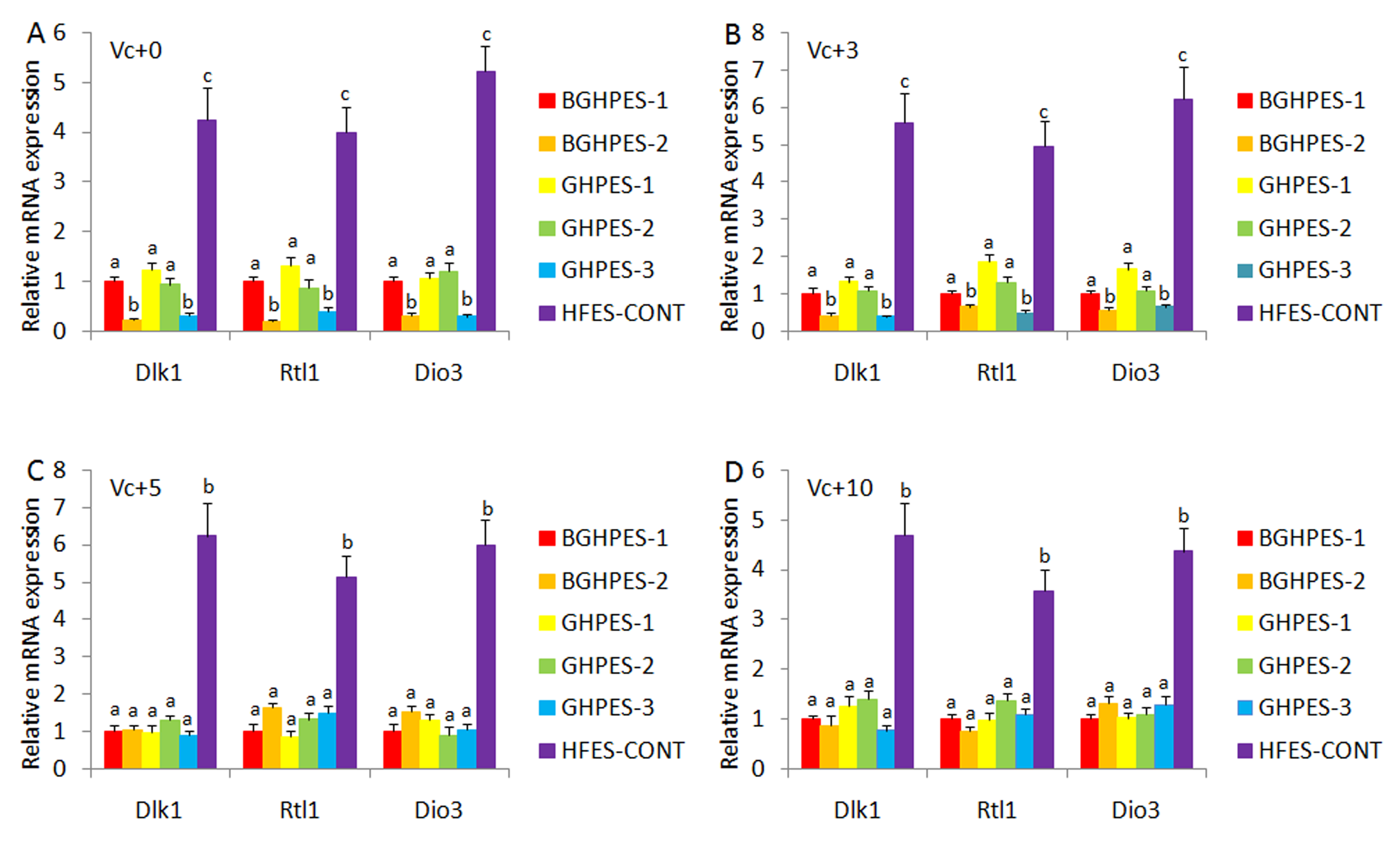

Supplement: Additional file 8: Figure S7. — Dynamic changes of paternally expressed genes in hpESCs after ascorbic acid treatment. In Vc + 0 (A) and Vc + 3 (B) groups, the expression level of Dlk1, Rtl1 and Dio3 was significantly lower in BGHPES-2 and GHPES-3, compared with BGHPES-1, GHPES-1 and GHPES-2; however, the expression level of these three genes in all five hpESCs lines was significantly lower than that in HFES-CONT. In Vc + 5 (A) and Vc + 10 (B) groups, the expression level of Dlk1, Rtl1 and Dio3 in BGHPES-2 and GHPES-3 was enhanced and comparable with BGHPES-1, GHPES-1 and GHPES-2; however, the expression level of these three genes in all five hpESCs lines was still significantly lower than that in HFES-CONT. Different letters indicate significant differences among data (P <0.05) while the same letters are used to signify no differences (P >0.05). [file 13287_2015_54_MOESM8_ESM.tiff]

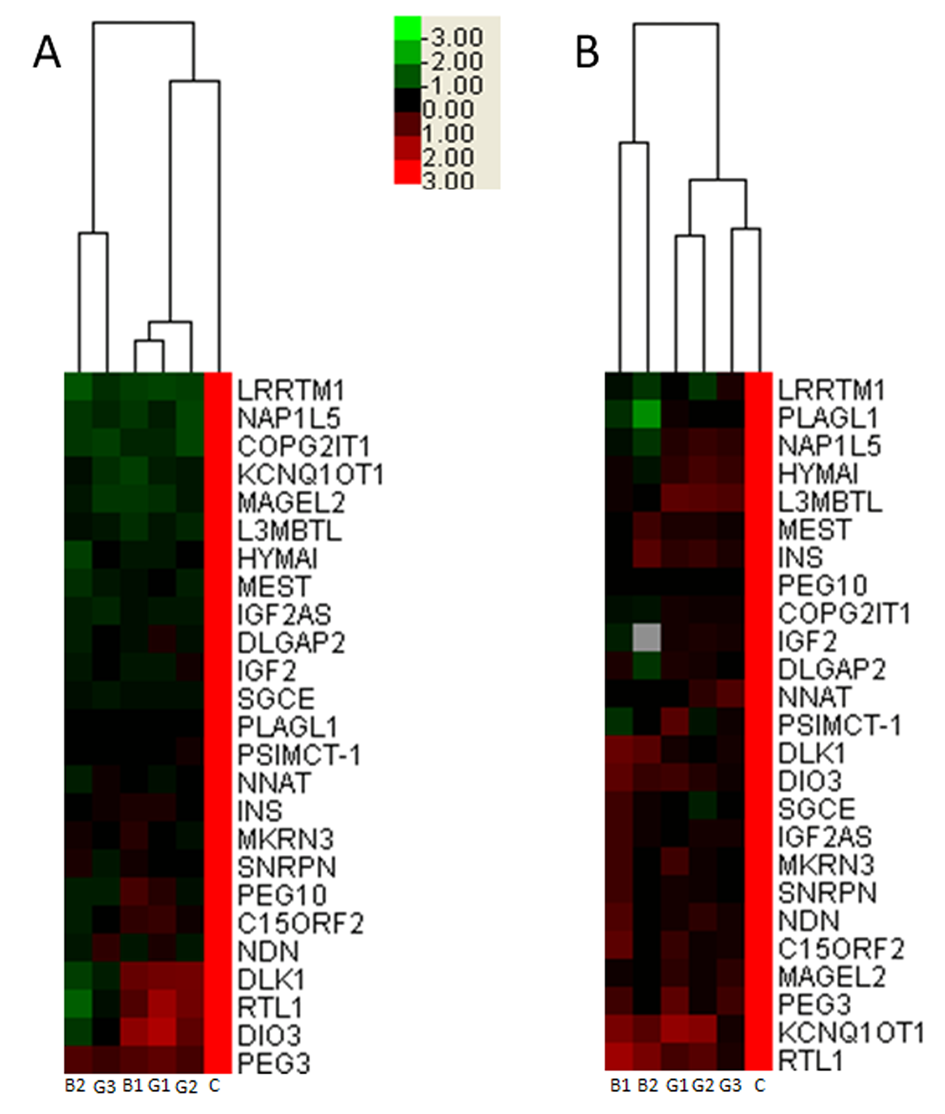

Supplement: Additional file 9: Figure S8. — Cluster analysis of paternally expressed genes in human parthenogenetic embryonic stem cells. (A) Differences in expression of Dlk1, Rtl1 and Dio3 between human parthenogenetic embryonic stem cells that form teratoma and those that do not form teratoma were observed. (B) No regular changes in Dlk1, Rtl1 and Dio3 were evident in human parthenogenetic embryonic stem cells after ascorbic acid treatment. [file 13287_2015_54_MOESM9_ESM.tiff]

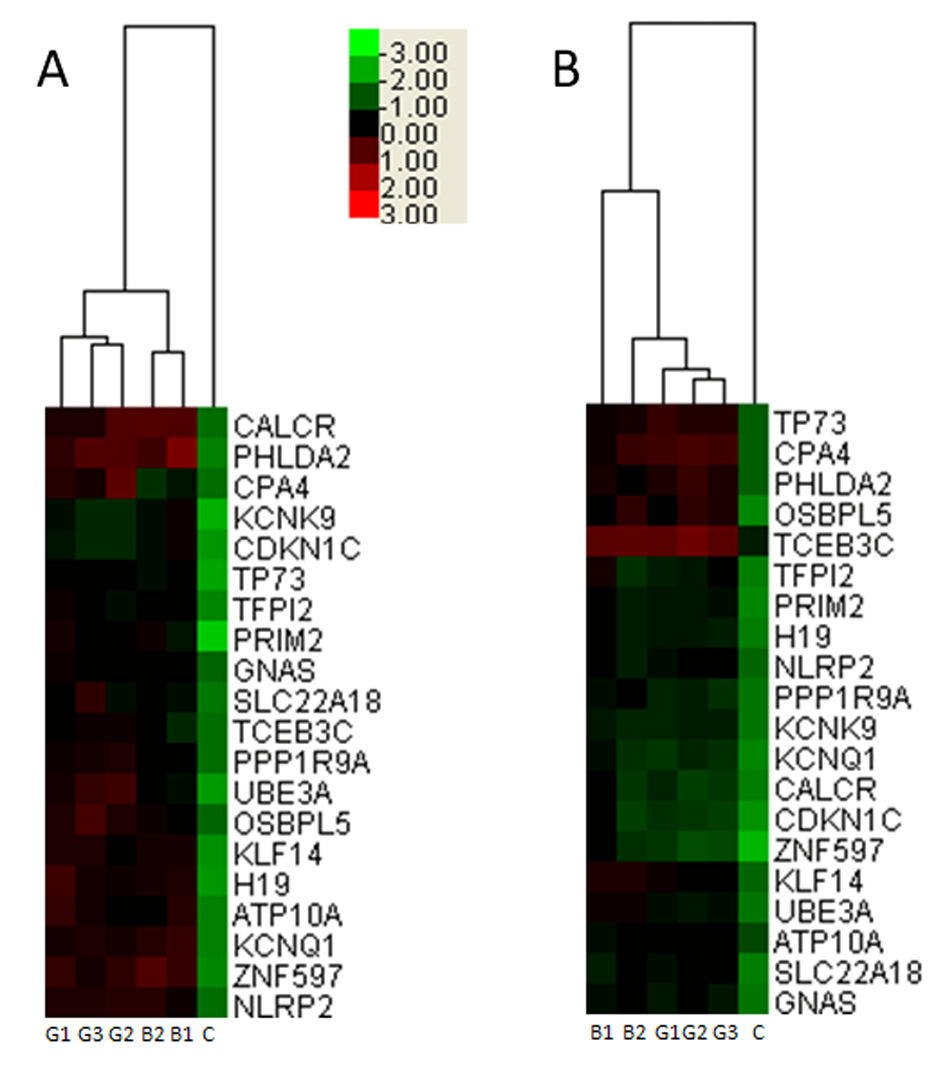

Supplement: Additional file 10: Figure S9. — Cluster analysis of maternally expressed genes in human parthenogenetic embryonic stem cells. No regular changes in 20 maternally expressed imprinting genes were evident in human parthenogenetic embryonic stem cells before (A) or after (B) ascorbic acid treatment. [file 13287_2015_54_MOESM10_ESM.tiff]
